# The Association Between Maternal Shaking Behavior and Inappropriate Infant Parenting: The Japan Environment and Children's Study

**DOI:** 10.3389/fpubh.2022.848321

**Published:** 2022-04-12

**Authors:** Aya Sakakihara, Toshio Masumoto, Youichi Kurozawa

**Affiliations:** ^1^Community Health Nursing, Faculty of Medicine, Shimane University, Shimane, Japan; ^2^Division of Health Administration and Promotion, Department of Social Medicine, Faculty of Medicine, Tottori University, Tottori, Japan

**Keywords:** Japan environment and children's study, shaken baby syndrome, child abuse, neglect, inappropriate parenting

## Abstract

**Background:**

Although many studies have identified risk factors for maternal shaking behavior, it is unknown whether mothers who have shaken their infants repeat shaking behavior or show other inappropriate parenting behaviors. Using data from the Japan Environment and Children's Study (JECS) birth cohort study, we investigated the associations between continuous shaking behavior and the associations between shaking behavior and other inappropriate parenting behaviors.

**Methods:**

JECS data starting from 2011 were used. Logistic regression was used to perform a cross-sectional analysis. The explanatory variable was shaking behavior and the dependent variables were leaving the infant home alone and hitting the infant (both at 1 month postpartum), and non-vaccination and infant burns (both at 6 months postpartum). A longitudinal analysis using logistic regression was also performed; here the explanatory variable was shaking behavior at 1 month postpartum and the dependent variables were shaking behavior, non-vaccination of the infant, and infant burns (all at 6 months postpartum).

**Results:**

In this study, 16.8% and 1.2% of mothers reported shaking behavior at 1 month and 6 months postpartum, respectively. Mothers who shook their infants at 1 month postpartum were approximately five times more likely to shake them at 6 months postpartum compared with mothers who had not shown previous shaking behavior (OR = 4.92, 95% CI [4.22, 5.73], *p* < 0.001). In Cross-sectional study, there were associations between shaking behavior and inappropriate parenting behavior such as hitting the infant and infant burns.

**Conclusion:**

The findings suggest that mothers who report early shaking behavior tend to subsequently repeat this behavior, and that shaking behavior may be associated with other inappropriate parenting behaviors.

## Introduction

Shaken baby syndrome (SBS), which is a type of abusive behavior, is caused by violent shaking of an infant and can lead to physical or mental disabilities (1). Its cardinal features are subdural hematoma, cerebral edema, and retinal hemorrhage (2). Shaking an infant is often triggered by excessive crying, mainly occurs in the first 3 to 6 months of an infant's life (2, 3) and was associated with infant factors (male sex) and maternal factors (primipara, postpartum depression, unwanted pregnancy, and young age) (4–8). Although some studies have retrospectively analyzed cases of abusive head trauma, including SBS (9–11), they have focused on medical outcomes such as subdural hematoma. Because medical outcome can only capture the small portion of violent shaking of an infant, it is important for prevention of abusive head trauma that epidemiological study about the head shaking behavior in general population is performed.

In a study based on a stratified regional sample in the Netherlands, the reported prevalence rates of SBS (3) were 1.01 at age 1 month, 1.32 at age 3 months, and 3.35% at age 6 months. Although studies conducted in Aichi and Chiba prefectures in Japan reported between 2.0 and 3.9% prevalence of SBS at 3–4 months of age (4, 5, 12), these studies were limited to specific geographical areas. Further studies with larger samples are needed to determine the prevalence of SBS in Japan more accurately.

Inappropriate parenting behaviors, including abusive behavior, have been shown to recur in the range of 20–60% depends on follow-up period (13, 14). Especially, the younger the child, the higher the recurrence rate of inappropriate parenting (14, 15). Therefore, although it is hypothesized that the risk of recurrence of SBS occurring in the infant is high, there were no studies about mothers who perform shaking behavior to their infants has tendency to repeat the shaking behavior. In addition, there were no studies about the association between shaking behavior and inappropriate behaviors such as leaving the infant home alone, hitting the infant, non-vaccination, or infant burns. To address this question, we used data from a birth cohort study, the Japan Environment and Children's Study (JECS), to investigate whether mothers who shook their infants continued to perform shaking behaviors or repeatedly showed other inappropriate parenting behaviors. We also examined which inappropriate parenting behaviors were associated with shaking behavior. These results will provide important insights into strategies for the prevention of outbreaks and recurrence of SBS and inappropriate parenting.

## Materials and Methods

### Study Design and Data Sources

The aim of the JECS, an ongoing prospective birth cohort study that began in 2011, is to evaluate the effect of various environmental factors on children's health and development (16, 17). The JECS Protocol was reviewed and approved by the Ministry of the Environment's Institutional Review Board on Epidemiological Studies and the Ethics Committees of all participating institutions. The present study is based on a JECS dataset (jecs-ta-20190930) released in October 2019. This dataset does not contain any patient identifying information. Written informed consent was obtained from all study participants.

The JECS protocol has been published elsewhere (16, 17). The JECS comprises a cohort of 104,062 children born from 2011 to 2014 in 15 Regional Centers covering 19 prefectures across Japan. We excluded the data of stillborn infants (3,758 pregnancies) and multiple pregnancy mothers (1,891 pregnancies) based on medical records. We excluded multiple pregnancy mothers because the same mother was registered with multiple infants in these cases, which led to duplicate data that made it difficult to evaluate shaking and inappropriate parenting behaviors due to parenting behavior to infants at the same time. Our dataset comprised 98,413 single live births. Questionnaires at 1 month postpartum were either sent and returned by mail or hand-delivered and then returned by hand or by mail. Questionnaires at 6 months postpartum were sent and returned by mail.

### Assessment of Shaking

We evaluated shaking using the following questions: “Frequency of shaking your baby very hard when he/she cries” from the JECS 1 month postpartum questionnaire and “Shook the child very hard in the past month” from the JECS 6 months postpartum questionnaire. Response options on the 1 month postpartum questionnaire were “Always,” “Sometimes,” “Seldom,” and “Not at all.” We considered responses of “Always,” “Sometimes,” and “Seldom” to indicate the presence of shaking behavior. Response options on the 6 months postpartum questionnaire were “None,” “Once,” “2–3 times,” and “4 or more times.” We considered responses of “Once,” “2–3 times,” and “4 or more times” to indicate the presence of shaking behavior.

### Assessment of Inappropriate Parenting Behaviors

We evaluated inappropriate parenting behaviors using the following questions: “Frequency of leaving the baby alone at home” and “Frequency of hitting the baby” from the 1 month postpartum questionnaire. Response options were “Always,” “Frequently,” “Sometimes,” “Seldom,” and “Not at all.” We considered responses of “Always,” “Frequently,” “Sometimes,” and “Seldom” to indicate the presence of inappropriate parenting behavior.

It has been reported an association between parental refusal of childhood vaccination and medical neglect (18); therefore, we used non-vaccination of the infant to measure inappropriate parenting behavior at 6 months postpartum. This was evaluated using “Vaccination received: None.” If the mother answered, “yes,” we assumed inappropriate parenting behavior. Because burns in young children often have a background of abuse or neglect (19), we evaluated abuse or neglect using the question, “Has your child been diagnosed with a disease (by a doctor) after birth? (check all that apply).” If the mother checked “burn” in response to this question, we assumed inappropriate parenting behavior.

### Covariates

Based on a previous study on child abuse (including SBS) (1), the following items were used as covariates: fatherless household (Yes, No), maternal age at delivery (older: ≥35 years; younger: ≤19 years; other: 20–34 years) (4–6, 20–24), household income (≥2 million yen; <2 million yen; about twenty thousand US dollars) (1, 4, 20, 25, 26), and postnatal depression using Edinburgh Postnatal Depression Scale at 1 month postpartum and 6 months postpartum (with postnatal depression: EPDS ≥ 9; without postnatal depression: <9) (4, 22, 27–29). The following items were used as covariates associated with only SBS: excessive crying (Yes or No) (1, 3, 4), type of nutrition (breastfeeding only, formula feeding only, or both formula and breastfeeding) (4), sex of child (male or female) (1, 6, 7), number of deliveries (primipara or multipara) (4). Because risk factors for excessive infant crying, which trigger SBS, include maternal education level (junior high school graduation or less, high school graduation or more) and domestic violence during pregnancy (with or without) (30), we additionally included these variables when using 6-month shaking behavior as an outcome.

### Statistical Analysis

A cross-sectional analysis using logistic regression was performed on the 1 month postpartum data. Responses to the questions about leaving the infant home alone and hitting the infant were the dependent variables, and violently shaking the infant was the explanatory variable; the analysis included the abovementioned covariates. A cross-sectional analysis using logistic regression was performed on the 6 months postpartum data, where non-vaccination and infant burns were the dependent variables and violently shaking the infant was the explanatory variable; the analysis included the abovementioned covariates. Univariate logistic regression analysis was initially performed, followed by multivariate logistic regression analysis for three models: Model 1, which included the variables fatherless household and young maternal age; Model 2, which included household income as an additional variable; and Model 3, which included postnatal depression as yet another additional variable.

A longitudinal analysis using logistic regression was also performed. Here, infant burns, and non-vaccination at 6 months postpartum were the dependent variables, and SBS at 1 month postpartum was the explanatory variable; the analysis included the abovementioned covariates. Univariate logistic regression analysis was initially performed, followed by multivariate logistic regression analysis for three models: Model 1, which included the variables fatherless household and young maternal age; Model 2, which included household income as an additional variable; and Model 3, which included postnatal depression as yet another additional variable.

For the longitudinal analysis of SBS at 1 month and 6 months postpartum, univariate logistic regression analysis was initially performed, followed by multivariate logistic regression analysis for three models: Model 1, which included fatherless household, primipara, maternal education and maternal age; Model 2, which included infant male sex and lower household income as additional variables; and Model 3, which included postnatal depression, type of nutrition (e.g., bottle-feeding in children aged 1 month), domestic violence during pregnancy and excessive crying at 1 month as further additional variables.

IBM SPSS Statistics 23 was used for the analysis, with the significance level set at <5%.

## Results

In this study, 16.8 and 1.2% mothers reported that they shook their infants very hard at 1 month postpartum and 6 months postpartum, respectively. Additionally, 18.3% of mothers reported excessive crying at 1 month postpartum (Table 1).

**Table 1:** Participant characteristics.

	**Total**	
	***N***	**%**
**Violently shaking infant aged 1 month (*****n*** **=** **95,641)**		
Yes (Always, Sometimes, Seldom)	16,508	16.8
No (Not at all)	79,133	80.4
**Violently shaking infant aged 6 months (*****n*** **=** **91,386)**		
Yes (more than 1 times in the last month)	1,158	1.2
No (Not at all)	90,228	91.7
**Sex of infant (*****n*** **=** **98,083)**		
Male	50,266	51.1
Female	47,817	48.6
**Maternal age (*****n*** **=** **77,505)**		
Younger (≤19 years)	697	0.7
Other (20–34 years)	55,599	56.5
Older (≥35 years)	21,209	21.6
**Postnatal depression at 1 month postpartum (*****n*** **=** **94,647)**		
Edinburgh postnatal depression scale score (≥9)	13,430	13.6
Edinburgh postnatal depression scale score (<9)	81,217	82.5
**Postnatal depression at 6 months postpartum (*****n*** **=** **90,309)**		
Edinburgh postnatal depression scale score (≥9)	10,526	10.7
Edinburgh postnatal depression scale score (<9)	79,783	81.1
**Excessive crying in infants aged 1 month (*****n*** **=** **95,525)**		
Yes	17,967	18.3
No	77,558	78.8
**Going out without the infant aged 1 month (*****n*** **=** **95,551)**		
Yes	15,020	15.3
No	80,531	81.8
**Hitting the infant aged 1 month (*****n*** **=** **95,686)**		
Yes	926	0.9
No	94,760	96.3
**Non-vaccination of infants aged 6 months (*****n*** **=** **91,710)**		
Yes	796	0.8
No	90,914	92.4
**Infant burns at 6 months (*****n*** **=** **91,710)**		
Yes	190	0.2
No	91,520	93.0

*N = 98,413*.

The univariate logistic regression cross-sectional analysis showed a significant association between infant shaking at 1 month postpartum and both leaving the infant home alone (odds ratio [OR], 1.47; 95% confidence interval [CI], 1.41–1.54), and hitting the infant (OR, 8.35; 95% CI, 7.30–9.56). The multivariate logistic regression also showed a significant association between shaking behavior at 1 month and the following variables: leaving the infant home alone (adjusted OR, 1.46; 95% CI, 1.38–1.53), and hitting the infant (adjusted OR, 6.60; 95% CI, 5.60–7.77) (Table 2).

**Table 2:** Association between shaking and inappropriate parenting behavior at 1 month postpartum: odds ratios and 95% confidence intervals.

**Outcome**		**Leaving the infant home alone at 1 month**	**Hitting the infant at 1 month**
Crude	OR (95% CI); P	1.47 (1.41–1.54); <0.001	8.35 (7.30–9.56); <0.001
Model 1^a^	OR (95% CI); P	1.49 (1.42–1.57); <0.001	7.76 (6.66–9.03); <0.001
Model 2^b^	OR (95% CI); P	1.48 (1.41–1.56); <0.001	7.74(6.60–9.08); <0.001
Model 3^c^	OR (95% CI); P	1.46(1.38–1.53); <0.001	6.60 (5.60–7.77); <0.001

*OR, odds ratio; CI, confidence interval*.

a *Model 1, adjusted for fatherless household and maternal age*.

b *Model 2, Model 1 + adjusted for household income*.

c *Model 3, Model 2 + adjusted for postnatal depression*.

The univariate logistic regression analysis showed a significant association between infant burns and shaking at 6 months postpartum (OR, 3.04; 95% CI, 1.43–6.49). There was no association between non-vaccination of infants and shaking at 6 months postpartum (OR, 1.51; 95% CI, 0.90–2.53). The same results were found for the multivariate regression analysis (adjusted OR for burns, 2.72; 95% CI, 1.10–6.71; adjusted OR for non-vaccination, 1.40; 95% CI, 0.78–2.50) (Table 3). These results indicate that there were the associations between shaking behavior and other inappropriate behaviors such as leaving the infant home alone, hitting the infant and infant burns that occurred at the same time.

**Table 3:** Association between shaking and inappropriate parenting behavior at 6 months postpartum: odds ratios and 95% confidence intervals.

**Outcome**		**Non-vaccination at 6 months**	**Infant burns at 6 months**
Crude	OR (95% CI); P	1.51 (0.90–2.53); 0.116	3.04 (1.43–6.49); 0.004
Model 1^a^	OR (95% CI); P	1.62 (0.93–2.82); 0.088	3.41 (1.50–7.75); 0.003
Model 2^b^	OR (95% CI); P	1.55 (0.87–2.77);0.137	2.93 (1.19–7.20); 0.019
Model 3^c^	OR (95% CI); P	1.40 (0.78–2.50); 0.260	2.72 (1.10–6.71); 0.030

*OR, odds ratio; CI, confidence interval*.

a *Model 1, adjusted for fatherless household and maternal age*.

b *Model 2, Model 1 + adjusted for household income*.

c *Model 3, Model 2 + adjusted for postnatal depression*.

To investigate the association between shaking behavior at 1 month postpartum and shaking behavior at 6 months, we performed a longitudinal analysis. Shaking behavior at 1 month postpartum was significantly associated with infant shaking at 6 months postpartum (OR, 5.80; 95% CI, 5.15–6.52). The multivariate logistic regression analysis of shaking behavior at 1 month postpartum and both shaking behavior produced the same results: for shaking behavior at 6 months postpartum (adjusted OR, 4.92; 95% CI, 4.22–5.73 (Table 4).

**Table 4:** Association between shaking at 1 month postpartum and shaking at 6 months postpartum: odds ratios and 95% confidence intervals.

**Outcome**		**Shaking at 6 months**
Crude	OR (95% CI); P	5.80 (5.15–6.52); <0.001
Model 1^a^	OR (95% CI); P	5.34 (4.64–6.14); <0.001
Model 2^b^	OR (95% CI); P	5.31 (4.59–6.15); <0.001
Model 3^c^	OR (95% CI); P	4.92 (4.22–5.73); <0.001

*OR, odds ratio; CI, confidence interval*.

a *Model 1, adjusted for fatherless household, primipara, maternal education and maternal age*.

b *Model 2, Model 1 + adjusted for sex of infant and household income*.

c *Model 3, Model 2 + adjusted for postnatal depression, type of nutrition in infants aged 1 month, domestic violence during pregnancy and excessive crying in infants aged 1 month*.

To elucidate the association between shaking behavior at 1 month postpartum and inappropriate parenting behaviors (such as non-vaccination and infant burns) at 6 months postpartum, we performed logistic regression analysis. Shaking behavior at 1 month postpartum was not associated with non-vaccination at 6 months (OR, 1.00; 95% CI, 0.83–1.20; adjusted OR, 1.00; 95% CI, 0.80–1.24) or infant burns at 6 months (OR, 1.18; 95% CI, 0.83–1.70; adjusted OR, 1.12; 95% CI, 0.73–1.72) (Table 5). These results indicate that there was association between shaking behavior to their infants at 1 month postpartum and shaking behavior at 6 months postpartum but no other inappropriate parenting behaviors at 6 months.

**Table 5:** Association between shaking at 1 month postpartum and inappropriate parenting behavior at 6 months postpartum: odds ratios and 95% confidence intervals.

**Outcome**		**Non-vaccination at 6 months**	**Infant burns at 6 months**
Crude	OR (95% CI); P	1.00 (0.83–1.20); 0.971	1.18 (0.83–1.70); 0.357
Model 1^a^	OR (95% CI); P	1.06 (0.86–1.30); 0.603	1.15 (0.76–1.74); 0.498
Model 2^b^	OR (95% CI); P	1.03 (0.83–1.29); 0.762	1.18 (0.78–1.80); 0.435
Model 3^c^	OR (95% CI); P	1.00 (0.80–1.24); 0.967	1.12 (0.73–1.72); 0.602

*OR, odds ratio; CI, confidence interval*.

a *Model 1, adjusted for fatherless household and maternal age*.

b *Model 2, Model 1 + adjusted for household income*.

c *Model 3, Model 2 + adjusted for postnatal depression*.

## Discussion

To our knowledge, this is the first study to investigate the prevalence of self-reported maternal shaking behavior using a representative Japanese sample, and to examine whether mothers who performed shaking behavior at 1 month postpartum continued this behavior or engaged in other inappropriate parenting behaviors at 6 months postpartum. The prevalence of self-reported shaking behavior at 1 month postpartum was 16.8%. The mothers who perform shaking behavior to infant at 1 month postpartum tend to perform shaking behavior at 6 months postpartum (Table 4). In addition, there were the associations between shaking behavior and other inappropriate behaviors that occurred at the same time (Tables 2, 3). However, we could see no association between shaking behavior to their infants at 1 month postpartum and other inappropriate parenting behaviors at 6 months postpartum except shaking behavior (Tables 4, 5). These results indicate that mothers who engaged in shaking behavior at 1 month postpartum tended to shake their infants at 6 months postpartum as well, in addition to performing other inappropriate parenting behaviors at 1 month and 6 months postpartum.

Previous studies have found a 1.01% prevalence of shaking behavior at 1 month postpartum in the Netherlands, and a 2.0–3.9% prevalence at 3–4 months postpartum in Japan (3–5, 12). The prevalence of shaking behavior seen in our study is very high in comparison. However, given that the peak of excessive infant crying is 5–6 weeks after birth (31), and that in our study 18.3% of infants exhibited excessive crying (a percentage similar to that of the 16.8% of shaking behavior), mothers may have experienced frustration and performed shaking behavior to stop their infants crying. On the other hand, at 6 months of age, crying decreases, which may indicate that shaking behavior has decreased. However, there is also evidence that shaking behavior is more likely to occur from 3–6 months postpartum (2, 3). Therefore, we might have found a higher prevalence of shaking behavior if we had conducted the study at 3–6 months postpartum. The sequelae of SBS include disorders of movement, vision, language, and behavior (32, 33). These SBS sequelae tend to have a poor prognosis in younger infants (33). Taken together, our results suggest the importance of explaining the risks of SBS to parents to prevent SBS in the early stages of infant development. In explaining the risks of SBS to parents, it is necessary to carefully and clearly inform such mothers of the risks of shaking and what to do when their child cries violently, because low maternal education level is a risk factor for excessive infant crying, which triggers SBS (30).

The longitudinal analysis showed that although shaking at 1 month postpartum was not associated with infant burns or non-vaccination at 6 months postpartum, mothers who shook their infants at 1 month postpartum were approximately five times more likely to shake their infants at 6 months postpartum. A parent who reports even one act of shaking should receive guidance and ongoing monitoring to prevent the recurrence of this behavior.

The cross-sectional analysis showed that shaking behavior at 1 month postpartum was associated with the risk of hitting the infant (OR, 6.6) and leaving the infant home alone (OR, 1.5). Shaking behavior at 6 months postpartum was associated with the risk of infant burns (OR, 2.7), but not with non-vaccination. These results suggest that violent behavior like shaking an infant is associated more with abusive behavior, such as hitting and infant burns, than with neglect (e.g., non-vaccination or leaving the infant home alone). A previous study found that 6% of parents performed abusive behaviors (e.g., choking, slapping, or shaking) to stop excessive crying in 6-month-old infants (3). Therefore, shaking is more likely to be accompanied by other impulsive acts of aggression. This indicates the importance of monitoring other parenting behaviors in parents who engage in shaking behavior. In addition, excessive crying is the risk factor of SBS. Considering that domestic violence, maternal bonding disorder, and depression are risk factors of excessive infant crying (30), it is possible that those who engage in shaking behaviors have a family background and mental distress. Therefore, early and intensive support for distressed mothers may help prevent the development of excessive infant crying, associated shaking behavior, and inappropriate parenting.

We would like to discuss four limitations in our study. First, as both shaking behavior and inappropriate parenting were assessed using maternal self-reports, it is possible that these behaviors were either underestimated (by mothers hiding their behavior) or overestimated (by mothers evaluating their parenting behavior negatively). These biases may create artificial associations and additional bias due to an unwillingness to disclose. For example, a mother who conceals her shaking behavior tends not to report other inappropriate parenting behaviors. However, because our methodology was consistent with those of previous studies with regard to self-report instruments and the definition of shaking behavior (3–5, 12), it still provides a useful comparison to previous prevalence rates. Second, there were some differences between the shaking questions asked at 1 month and 6 months postpartum. Whereas the question at 1 month postpartum specifically mentioned shaking in response to crying, the question about shaking at 6 months postpartum was not limited in this way. However, we defined the presence of shaking behavior (one or more times) exactly the same way in both cases. We note that we could not clarify the reason about the decreasing of shaking behavior at 6 months postpartum compared with 1 month postpartum. To elucidate this, further study is needed. Third, although previous studies indicate that fathers are the most frequent perpetrators of abusive head trauma (8), our sample included only mothers. This may have resulted in an underestimation of shaking behavior. Additional studies are needed that include fathers. Fourth, since we analyzed the data collected by the JECS group promoting by the Ministry of the Environment, Japan, we could only conduct the survey using the limited items about inappropriate parenting. To elucidate the association between shaking behavior and parenting behavior, further study should be needed.

Despite these limitations, this study is the first that we know of to use a representative sample of mothers from across Japan. The results suggest that mothers with shaking babies at 1 month postpartum have tendency to shake their babies at 6 months and associate with inappropriate parenting behaviors. These findings may be important in the development of measures to prevent SBS, and in relapse prevention.

## Data Availability Statement

The data analyzed in this study was subject to the following licenses/restrictions: data are unsuitable for public deposition due to the ethical restrictions and legal framework of Japan. It is prohibited by the Act on the Protection of Personal Information (Act No. 57 of 30 May 2003, amendment on 9 September 2015) to publicly deposit data containing personal information. Ethical Guidelines for Medical and Health Research Involving Human Subjects enforced by the Japan Ministry of Education, Culture, Sports, Science and Technology, and the Ministry of Health, Labor and Welfare also restricts the open sharing of epidemiological data. All inquiries about access to data should be sent to: jecs-en@nies.go.jp. The person responsible for handling enquiries sent to this e-mail address is Shoji F. Nakayama, JECS Programme Office, National Institute for Environmental Studies. Requests to access these datasets should be directed to jecs-en@nies.go.jp.

## Ethics Statement

The studies involving human participants were reviewed and approved by The Ministry of the Environment's Institutional Review Board on Epidemiological Studies. Written informed consent to participate in this study was provided by the participants' legal guardian/next of kin.

## Author Contributions

AS and JECS Group were involved in study design and data interpretation. AS and TM were involved in the data analysis. YK and JECS Group were involved in data collection. All authors critically revised the report, commented on drafts of the manuscript, and approved the final report.

## Funding

The Japan Environment and Children's Study was funded by the Ministry of the Environment, Japan. The findings and conclusions of this article are solely the responsibility of the authors and do not represent the official views of the Japanese government.

## Conflict of Interest

The authors declare that the research was conducted in the absence of any commercial or financial relationships that could be construed as a potential conflict of interest.

## Publisher's Note

All claims expressed in this article are solely those of the authors and do not necessarily represent those of their affiliated organizations, or those of the publisher, the editors and the reviewers. Any product that may be evaluated in this article, or claim that may be made by its manufacturer, is not guaranteed or endorsed by the publisher.

## References

[1] Lopes NR Eisenstein E Williams LC. Abusive head trauma in children: a literature review. J Pediatr. (2013) 89:426–33. 10.1016/j.jped.2013.01.011 23850113

[2] Vinchon M. Shaken baby syndrome: what certainty do we have? Childs Nerv Syst. (2017) 33:1727–727:1727–33. 10.1007/s00381-017-3517-8 29149395

[3] Reijneveld SA van der Wal MF Brugman E Sing RA Verloove-Vanhorick SP. Infant crying and abuse. Lancet. (2004) 364:1340–2. 10.1016/S0140-6736(04)17191-2 15474137

[4] Fujiwara T Yamaoka Y Morisaki N. Self-reported prevalence and risk factors for shaking and smothering among mothers of 4-month-old infants in Japan. J Epidemiol. (2016) 26:4–13. 10.2188/jea.JE20140216 26639749 PMC4690735

[5] Isumi A Fujiwara T. Synergistic effects of unintended pregnancy and young motherhood on shaking and smothering of infants among caregivers in Nagoya city, Japan. Front Public Health. (2017) 5:245. 10.3389/fpubh.2017.00245 29018790 PMC5614918

[6] Keenan HT Runyan DK Marshall SW Nocera MA Merten DF Sinal SH. Population-based study of inflicted traumatic brain injury in young children. JAMA. (2003) 290:621–6. 10.1001/jama.290.5.621 12902365

[7] Fanconi M Lips U. Shaken baby syndrome in switzerland: results of a prospective follow-up study, 2002–2007. Eur J Pediatr. (2010) 169:1023–8. 10.1007/s00431-010-1175-x 20213304

[8] Scribano PV Makoroff KL Feldman KW Berger RP. Association of perpetrator relationship to abusive head trauma clinical outcomes. Child Abuse Negl. (2013) 37:771–7. 10.1016/j.chiabu.2013.04.011 23735871

[9] Datta S Stoodley N Jayawant S Renowden S Kemp A. neuroradiological aspects of subdural haemorrhages. Arch Dis Child. (2005) 90:947–51. 10.1136/adc.2002.021154 16113131 PMC1720557

[10] Trenchs V Curcoy AI Navarro R Pou J. Subdural haematomas and physical abuse in the first 2 years of life. Pediatr Neurosurg. (2007) 43:352–7. 10.1159/000106382 17785998

[11] Gerber P Coffman K. Non-accidental head trauma in infants. Child Nerv Sys. (2007) 23:499–507. 10.1007/s00381-006-0267-4 17370080

[12] Yamada F Fujiwara T. Prevalence of self-reported shaking and smothering and their associations with co-sleeping among 4-month-old infants in Japan. Int J Environ Res Public Health. (2014) 11:6485–93. 10.3390/ijerph110606485 25003171 PMC4078590

[13] Casanueva C Tueller S Dolan M Testa M Smith K Day O. Examining predictors of re-reports and recurrence of child maltreatment using two national data sources. Child Youth Serv Rev. (2015) 48:1–13. 10.1016/j.childyouth.2014.10.006

[14] Drake B Jonson-Reid M Sapokaite L. Rereporting of child maltreatment: does participation in other public sector services moderate the likelihood of a second maltreatment report?. Child Abuse Negl. (2006) 30:1201–26. 10.1016/j.chiabu.2006.05.008 17112587 PMC3562123

[15] Connell CM Bergeron N Katz KH Saunders L Tebes JK. Re-referral to child protective services: the influence of child, family, and case characteristics on risk status. Child Abuse Negl. (2007) 31:573–88. 10.1016/j.chiabu.2006.12.004 17537504

[16] Michikawa T Nitta H Nakayama S Yamazaki S Isobe T Tamura K. Baseline profile of participants in the Japan environment and children's study (JECS). J Epidemiol. (2018) 28:99–104. 10.2188/jea.JE20170018 29093304 PMC5792233

[17] Kawamoto T Nitta H Murata K Toda E Tsukamoto N Hasegawa M. Rationale and study design of the japan environment and children's study (JECS). BMC Public Health. (2014) 14:25. 10.1186/1471-2458-14-25 24410977 PMC3893509

[18] Parasidis E Opel D. Parental refusal of childhood vaccines and medical neglect laws. Am J Public Health. (2017) 107:68–71. 10.2105/AJPH.2016.303500 27854538 PMC5308147

[19] James-Ellison M Barnes P Maddocks A Wareham K Drew P Dickson W. Social health outcomes following thermal injuries: a retrospective matched cohort study. Arch Dis Child. (2009) 94:663–7. 10.1136/adc.2008.143727 19531525

[20] Sidebotham P Heron J. ALSPAC Study Team. Child maltreatment in the “children of the nineties”: a cohort study of risk factors. Child Abuse Negl. (2006) 30:497–522. 10.1016/j.chiabu.2005.11.005 16701895

[21] Ooki S. Fatal child maltreatment associated with multiple births in Japan: nationwide data between July 2003 and March 2011. Environ Health Prev. (2013) 16:416–21. 10.1007/s12199-013-0335-9 23558473 PMC3773100

[22] Yoshioka-Maeda K Kuroda M. Characteristics and related factors of japanese mothers who have faced difficulties with childrearing. Public Health Nurs. (2017) 34:422–9. 10.1111/phn.12328 28419536

[23] Yang YO Peden-McAlpine C Chen CH. A Qualitative study of the experiences of Taiwanese women having their first baby after the age of 35 years. Midwifery. (2007) 23:343–9. 10.1016/j.midw.2006.03.009 17229507

[24] Sonobe M Usui M Hiroi K Asai H Hiramatsu M Nekoda Y. Influence of older primiparity on childbirth, parenting stress, and mother-child interaction. Jpn J Nurs Sci. (2016) 13:229–39. 10.1111/jjns.12110 26782086

[25] Jabraeili M Asadollahi M Asghari Jafarabadi M Hallaj M. Attitude toward child abuse among mothers referring health centers of tabriz. J Caring Sci. (2015) 4:75–82. 10.17795/intjsh-31198 25821761 PMC4363654

[26] Nu198sh- Sci abuse among motherK Adamo MA Drazin D. Outcomes and factors associated with infant abusive head trauma in the US. J Neurosurg Pediatr. (2015) 16:515–22. 10.3171/2015.3.PEDS14544 26230462

[27] Windham AM Rosenberg L Fuddy L McFarlane E Sia C Duggan AK. Risk of mother-reported child abuse in the first 3 years of life. Child Abuse Negl. (2004) 28:645–67. 10.1016/j.chiabu.2004.01.003 15193853

[28] Cox JL Holden JM Sagovsky R. Detection of postnatal depression: development of the 10-item edinburgh postnatal depression scale. Br J Psychiatry. (1987) 150:782–6. 10.1192/bjp.150.6.782 3651732

[29] O0.6.782LM Heycock EG Hanna M Jones PW Cox JL. Postnatal depression and faltering growth: a community study. Pediatrics. (2004) 113:1242–7. 10.1542/peds.113.5.1242 15121936

[30] Yalçin SS Orün E Mutlu B Madendag Y Sinici I Dursun A. Why are they having infant colic? A nested case-control study. Perinat Epidemiol. (2010) 24:586–96. 10.1111/j.1365-3016.2010.01150.x 20955236

[31] Barr RG Trent RB Cross J. Age-Related incidence curve of hospitalized shaken baby syndrome cases: convergent evidence for crying as a trigger to shaking. Child Abuse Negl. (2006) 30:7–16. 10.1016/j.chiabu.2005.06.009 16406023

[32] Barlow KM Thomson E Johnson D Minns RA. Late Neurologic and cognitive sequelae of inflicted traumatic brain injury in infancy. Pediatrics. (2005) 116:e174–85. 10.1542/peds.2004-2739 16061571

[33] Talvik I Mequelae Mequelae of inflicted traumatic brain injury in . Outcome of infants with inflicted traumatic brain injury (shaken baby syndrome) in estonia. Acta Paediatr. (2007) 96:1164–164:1164–8. 10.1111/j.1651-2227.2007.00362.x 17578492

